# Imported scrub typhus: first case in South America and review of the literature

**DOI:** 10.1186/s40794-018-0070-8

**Published:** 2018-08-16

**Authors:** Thomas Weitzel, Mabel Aylwin, Constanza Martínez-Valdebenito, Ju Jiang, Jose Manuel Munita, Luis Thompson, Katia Abarca, Allen L. Richards

**Affiliations:** 1 Laboratorio Clínico, Clínica Alemana de Santiago, Facultad de Medicina Clínica Alemana, Universidad del Desarrollo, Av. Vitacura, 5951 Santiago, Chile; 2 Servicio de Infectología, Clínica Alemana de Santiago, Facultad de Medicina Clínica Alemana, Universidad del Desarrollo, Santiago, Chile; 3 Departamento de Enfermedades Infecciosas e Inmunología Pediátricas, Escuela de Medicina, Pontificia Universidad Católica de Chile, Santiago, Chile; 4 Viral and Rickettsial Diseases Department, Naval Medical Research Center, Silver Spring, MD USA; 5 Department of Preventive Medicine and Biostatistics, Uniformed Services University of the Health Sciences, Bethesda, MD USA

**Keywords:** Arthropod-borne diseases, Scrub typhus, *Orientia tsutsugamushi*, Travel, Imported infection

## Abstract

**Background:**

Scrub typhus is a neglected vector-borne zoonosis causing life-threatening illnesses, endemic in the Asian-Pacific region and, as recently discovered, in southern Chile. Scrub typhus is rarely reported in travelers, most probably due to the lack of clinical experience and diagnostic tests in non-endemic countries. We report the first case of imported scrub typhus in South America.

**Case presentation:**

A 62-year-old tourist from South Korea presented severely ill with fever, rash, and eschar in Santiago, Chile. Laboratory exams showed thrombocytopenia and elevated inflammation parameters, hepatic enzymes, and LDH. With the clinical suspicion of scrub typhus, empirical treatment with doxycycline was initiated and the patient recovered rapidly and without complications. The diagnosis was confirmed by IgM serology and by real-time PCR, which demonstrated infection with *Orientia tsutsugamushi* (Kawasaki clade).

**Conclusions:**

Only due to the emerging clinical experience with endemic South American scrub typhus and the recent implementation of appropriate diagnostic techniques in Chile, were we able to firstly identify and adequately manage a severe case of imported scrub typhus in South America. Physicians attending febrile travelers need to be aware of this rickettsiosis, since it requires prompt treatment with doxycycline to avoid complications.

## Background

Scrub typhus is a vector-borne zoonosis caused by *Orientia* species that manifests as an acute febrile disease and has a potentially severe outcome [1]. It is transmitted by the larval stage of trombiculid mites called ‘chiggers’. After the bite of an infective chigger, a characteristic necrotic inoculation lesion termed eschar might develop, which typically contains high bacterial loads. The microorganism then spreads via lymphatics and blood, causing systemic manifestations and laboratory abnormalities such as elevated C-reactive protein (CRP) and liver enzymes [1]. Although widely under-recognized, scrub typhus is considered the most important rickettsial infection worldwide threatening over a billion people and causing more than a million cases per year with substantial mortality [2]. Until recently, scrub typhus was associated with a single species, *O. tsutsugamushi*, which exclusively occurred within the so-called ‘Tsutsugamushi Triangle’ ranging from Pakistan in the West, far-eastern Russia in the East to northern Australia in the South. However, recent reports of autochthonous cases in the Middle East and southern Chile have reshaped this epidemiological paradigm, suggesting a wider geographical distribution [3, 4].

Scrub typhus is very rarely diagnosed in travelers, with a total of < 40 cases reported in the medical literature. Still, the problem might be under-recognized, since the initial clinical suspicion relies on the physicians’ experience, routine laboratory tests are largely unavailable, and only few reference laboratories permit a definite diagnosis by molecular methods and/or culture. Here we report an *Orientia tsutsugamushi* infection in a traveler from South Korea visiting Chile, which was confirmed by molecular methods and serology.

## Case presentation

**Fig. 1:**
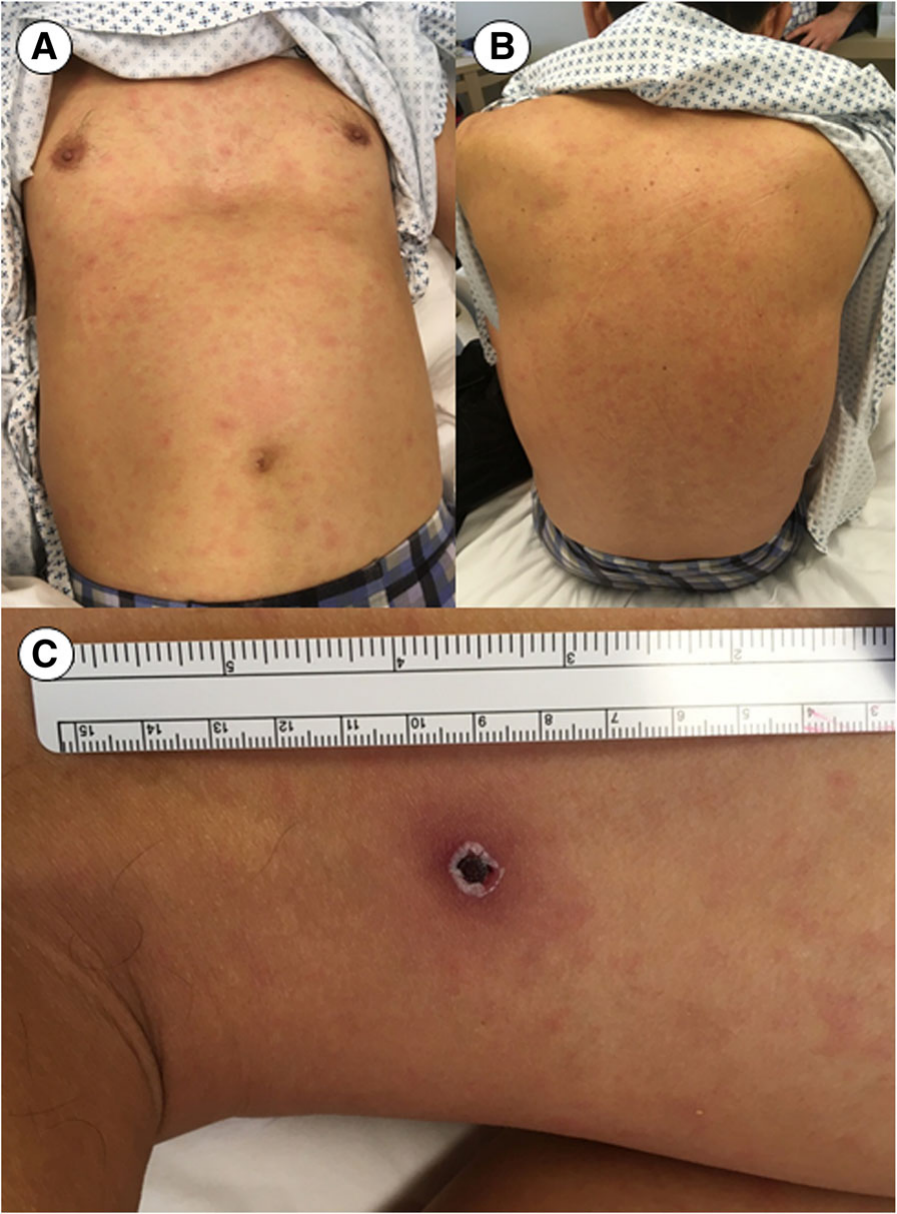
Coarse maculopapular rash of South Korean patient, predominantly affecting the trunk (**a**, **b**), accompanied by characteristic necrotic eschar on the dorsal face of the left thigh (**c**)

A 62-year-old South Korean tourist presented with fever, rash, and severe malaise two days after arrival in Chile. He reported flu-like symptoms with chills, headache, and myalgia, which had begun five days earlier. The patient was from Seoul, but had recently visited a farm south of Seoul. At presentation, the patient suffered intense headache and felt severely unwell. He was tachycardic (108 bpm), had an axillar temperature of 38.8 C, and a generalized, non-pruritic maculopapular rash, more pronounced on the trunk and sparing palms, soles, and mucosa (Fig. 1, A and B). On his left posterior thigh, there was a painless necrotic lesion with a diameter of 6 mm and a surrounding red halo (Fig. 1, C). Complete blood count showed mild thrombocytopenia (91,000/μL), low hemoglobin (13.0 g/dL), and normal leukocytes with a left shift (bands, 20%), toxic granulation, and atypical (reactive) lymphocytes. Other laboratory exams revealed elevated inflammation parameters (ESR, 32 mm/h; CRP, 3.6 mg/dL), slight hyponatremia (134 mEq/L), elevated hepatic enzymes (AST, 180 U/L; ALT, 161 U/L; GGT 327 U/L; AP, 208 U/L) and LDH (718 U/L). A molecular respiratory panel (xTAG® Respiratory Viral Panel FAST v2; Luminex, Austin, TX, USA) as well as serological assays (IgM) for EBV, CMV, and Parvovirus B19 were all negative.

**Fig. 2:**
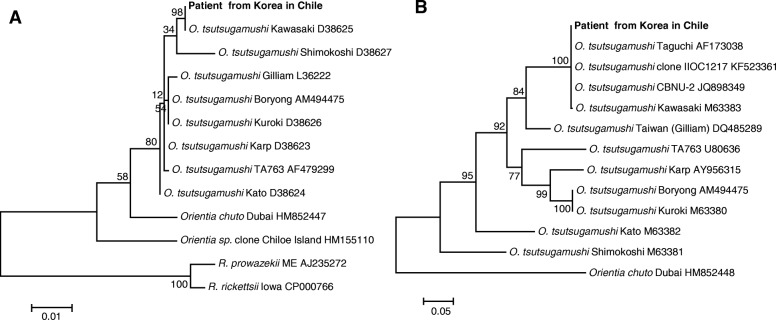
Phylogenetic analysis of *Orientia tsutsugamushi* isolate amplified from eschar sample of patient from South Korea. The trees were constructed based on 970 bp *rrs* (**a**) and 593 bp *tsa56* (**b**) gene fragments of the patient and *O. tsutsugamushi* type strains/isolates (GenBank accession numbers are shown next to each agent) using the Maximum Likelihood method with the Tamura-Nei model. Evolutionary analyses were conducted in MEGA7 and the values for the bootstrap test (1000 replicates) are shown next to the branches

Oral treatment with doxycycline (100 mg bid) was initiated in response to clinical suspicion of scrub typhus. Blood samples were drawn, the necrotic eschar was removed and placed in 70% alcohol, and a dry swab sample was taken from the base of the unroofed lesion. The following day, examination of serum by commercial ELISA (Scrub Typhus Detect, InBios International Inc., Seattle, WA, USA) was positive for IgM and negative for IgG antibodies. A commercial indirect IgG immunofluorescence test (Fuller Laboratories, Fullerton, CA, USA) utilizing acetone-fixed *O. tsutsugamushi* strains (Gilliam, Karp, Kato, Boryong) was positive at a dilution of 1:64 for the Karp antigen. DNA preparations from buffy coat as well as eschar material and swab samples were positive by quantitative real-time PCR (Otsu47) targeting the *O. tsutsugamushi*-specific 47kD protein gene (*htrA*) using primers and probe described previously [5]. From the eschar sample, *rrs, htrA* and 56 kDa type specific antigen (*tsa56*) gene fragments were successfully amplified using semi-nested PCR assays (Table 1). Amplicons were purified and sequenced by 3500 Genetic Analyzer (Thermo Fisher Scientific, Waltham, MA, USA), the sequences from each primer were assembled with CodonCode Aligner (CodonCode Corporation, Centerville, MA, USA), and the consensus sequence of 1045, 1456 and 1302 bp fragments were obtained for *rrs*, *htrA,* and *tsa56*, respectively. Sequences were deposited in GenBank (accession numbers MG844362 [*rrs*], MG844360 [*htrA*], and MG844361 [*tsa56*]). Blast search (https://blast.ncbi.nlm.nih.gov/Blast.cgi) revealed that the pathogen was closest to *O. tsutsugamushi* Kawasaki type strain, with 100% identity for *rrs* (*O. tsutsugamushi* Kawasaki) and *tsa56*, including isolates from Korea (CBNU-2 and IIOC1217) [6] and Japan (Taguchi) [7]. Phylogenetic analysis also showed that the isolate clustered with Kawasaki type strains for both *rrs* and *tsa56*, but was not related with the *Orientia* sp. Chiloe Island isolate recently discovered from Chile (Fig. 2).

**Table 1:** Primers and probes used for diagnostic and phylogenetic analysis

Primer ID	Sequence	Annealing temp.	PCR	Reference
16sU17F	AGAGTTTGATCCTGGCTCAG	56 °C	First	[30]
16sOR1198R	TTTCCTATAGTTCCCGGCATT	56 °C	First & second	
16sO79F	ATTAATGCTGAGCTTGCTTAGCAT	56 °C	Second	
Otr47_263F	GTGCTAAGAAARGATGATACTTC	54 °C	First	[31]
Otr1780R	AAATCGCCTTTAAACTAGATTTACTTATTA	54 °C	First & second	
Otr47F	TAAAGGTTAAGTTTATGAAAAAGGCATTT	54 °C	Second	
Otr56_498F	AATTAGTTTAGAATGGTTACCAC	54 °C	First	
r56_585F	AATGTCTGCGTTGTCGTTGC	54 °C	Second	
r56_2057	TCCACATACACACCTTCAGC	54 °C	First & second	[32]
OtsuFP630	AACTGATTTTATTCAAACTAATGCTGCT	60 °C	Real time	[5]
OtsuRP747	TATGCCTGAGTAAGATACRTGAATRGAATT	60 °C	Real time	[5]
OtsuPR665	6FAM-TGGGTAGCTTTGGTGGACCGATGTTTAATCT-TAMRA	60 °C	Real time	[5]

With antibiotic treatment, fever subsided within 36 h and the patient was discharged after three days.

## Discussion

Although scrub typhus represents an important public health issue in the Asia-Pacific region and is one of the most severe rickettsial infections, it is almost unrecognized in travelers and poorly covered in web-based information platforms and textbooks of Travel Medicine. A review from 2004 includes ~ 20 cases in travelers [8]; since then, < 15 additional patients have been published, all as single case reports or small case series [9–19]. The GeoSentinel network reported only five confirmed cases among 47,915 ill travelers between 1996 and 2008 [20]. Most of these patients were diagnosed by serological tests of single (mostly convalescent) samples and only few were molecularly confirmed (mostly in endemic countries) [18, 19]. In contrast, many experts postulate an increased risk of travel-associated scrub typhus due to the emergence of ecotourism (camping, trekking, rafting) in endemic areas [8, 21, 22], which is in accordance with experiences during military operations during World War II and the Vietnam and Korea conflicts, when scrub typhus affected thousands of soldiers [23]. The main reasons for the sustained paucity of reports in Travel Medicine, is most probably the lack of clinical experience and diagnostic tools in many non-endemic countries. In South America, for example, diagnostic tests for scrub typhus were unavailable until recently, and imported cases have never been described. In our case, the emerging clinical experience with endemic South American scrub typhus [3] and recent implementation of diagnostic techniques in Chile permitted the proper management and rapid diagnostic confirmation of this imported case.

Routinely, scrub typhus is diagnosed by serology, either by positive IgM or IgG seroconversion; though early cases are often seronegative. Definitive diagnosis mostly relies on molecular methods, preferably from eschar material, which stays positive even after initiation of treatment [24]. As in other severe rickettsial infections, empirical treatment should never be delayed due to diagnostic difficulties. In our case, PCR and serology permitted a timely (but also retrospective) diagnosis, proving infection with *O. tsutsugamushi* Kawasaki strain, which was acquired in the Imsil district, a region of high scrub typhus incidence in South Korea [25].

Untreated scrub typhus has a high rate of complications and mortality, especially in naïve patients [21]. The immediate start of appropriate antibiotic treatment is therefore the main goal of clinical management and lowers the risk of severe manifestations. For this, the physicians’ clinical experience and judgment is crucial. The most characteristic sign of scrub typhus is a necrotic skin lesion (eschar) at the inoculation site. It appears in 20–90% of patients, but seems to be more frequently present in naïve patients, e.g. travelers [1, 22]. To detect the inconspicuous and painless lesion, a thorough physical exam is essential. If the eschar is not detected or patients do not present the lesion, diagnosis is often delayed and complications more probable. Scrub typhus may also affect domestic travelers. This phenomenon has been recognized in Asian countries such as Taiwan [26], and is also an emerging problem in Chilean travelers returning to the central metropolitan region from trips to endemic regions in southern Chile (unpublished data). The main differential diagnoses in eschar-positive febrile travelers are spotted fever group rickettsioses. In our patient, those included Japanese spotted fever caused by *Rickettsia japonica* and other endemic rickettsiae endemic in South Korea such as *R. monacensis, R. felis,* and *R. akari* [27, 28]. The centrifugal distribution of the rash, sparing palms and soles, was suggestive for scrub typhus, since rickettsial spotted fevers have a centripetal rash including palms and soles [29]. In comparison to the rash of patients with dengue or other arboviral infections, the rash in scrub typhus has a coarser and more irregular appearance. The presence of atypical lymphocytes might lead to the misdiagnosis of infective mononucleosis, especially in the presence of lymphadenopathy.

## Conclusions

This first case of imported scrub typhus in South America highlights the need of physicians attending febrile travelers to be aware of this severe rickettsiosis. This includes knowledge of the endemic regions in Asia, its emergence in South America, and the recognition of the typical clinical manifestations. As in other rickettsial infections, rapid clinical recognition and prompt empirical treatment with doxycycline are crucial for a favorable patient’s prognosis. Diagnostic tools, ideally molecular methods, are useful for retrospective confirmation, but are only available in specialized laboratories. Pre-travel consultations should include information on the risk and prevention of this infection, especially for travelers with outdoor activities such as camping and trekking in endemic regions.
